# Parameter estimation in biochemical systems models with alternating regression

**DOI:** 10.1186/1742-4682-3-25

**Published:** 2006-07-19

**Authors:** I-Chun Chou, Harald Martens, Eberhard O Voit

**Affiliations:** 1 The Wallace H. Coulter Department of Biomedical Engineering at Georgia Institute of Technology and Emory University, 313 Ferst Drive, Atlanta, GA, 30332, USA; 2 CIGENE/Norwegian U. of Life Sciences, P.O.Box 5003, N – 1432 Ås, Norway

## Abstract

**Background:**

The estimation of parameter values continues to be the bottleneck of the computational analysis of biological systems. It is therefore necessary to develop improved methods that are effective, fast, and scalable.

**Results:**

We show here that *alternating regression *(AR), applied to S-system models and combined with methods for decoupling systems of differential equations, provides a fast new tool for identifying parameter values from time series data. The key feature of AR is that it dissects the nonlinear inverse problem of estimating parameter values into iterative steps of linear regression. We show with several artificial examples that the method works well in many cases. In cases of no convergence, it is feasible to dedicate some computational effort to identifying suitable start values and search settings, because the method is fast in comparison to conventional methods that the search for suitable initial values is easily recouped. Because parameter estimation and the identification of system structure are closely related in S-system modeling, the AR method is beneficial for the latter as well. Specifically, we show with an example from the literature that AR is three to five orders of magnitudes faster than direct structure identifications in systems of nonlinear differential equations.

**Conclusion:**

Alternating regression provides a strategy for the estimation of parameter values and the identification of structure and regulation in S-systems that is genuinely different from all existing methods. Alternating regression is usually very fast, but its convergence patterns are complex and will require further investigation. In cases where convergence is an issue, the enormous speed of the method renders it feasible to select several initial guesses and search settings as an effective countermeasure.

## Background

Novel high-throughput techniques of molecular biology are capable of producing *in vivo *time series data that are relatively high in quantity and quality. These data implicitly contain enormous information about the biological system they describe, such as their functional connectivity and regulation. The hidden information is to be extracted with methods of parameter estimation, if the structure of the system is known, or with methods of structure identification, if the topology and regulation of the system are not known. The S-system format within Biochemical Systems Theory (BST; [1-4]) is recognized as a particularly effective modeling framework for both tasks, since it has a mathematically convenient structure and because every parameter has a uniquely defined meaning and role in the biological system. Due to the latter feature, the typically complex identification of the pathway structure reduces to a parameter estimation task, though in a much higher-dimensional space. Still, like most other biological models, S-system models are nonlinear, so that parameter estimation is a significant challenge. Here, we propose a method called *alternating regression *(AR), which we combine with a previously described decoupling technique [5]. AR is fast and rather stable, and performs structure identification tasks between 1,000 and 50,000 times faster than methods that directly estimate systems of nonlinear differential equations (*cf*. [6]).

## Methods

### Modeling framework

In the S-system formulation within BST, *X*_*i *_denotes the concentration of metabolite *i*, and its change over time, , is represented as the difference between one production and one degradation term, both of which are formulated as products of power-law functions.*

(* Footnote: Throughout the paper, metabolite concentrations are represented as upper-case italics (*X*). An upper-case boldface variable (**L**) represents a matrix of regressor columns and a lower-case boldface variable (**y**) represents a regressand column in a linear multivariate statistical regression model.)

The generic form of an S-system is thus



The rate constants *α*_*i *_and *β*_*i *_are non-negative and the kinetic orders *g*_*ij *_and *h*_*ij *_are real numbers with typical values between -1 and +2. The S-system format allows the inclusion of independent variables, but because these are typically known in estimation tasks and constant, they can be merged with the rate constants [4]. S-systems have been discussed many times [3,4,7,8] and need no further explanations here.

### Decoupling of differential equations

Suppose the S-system consists of *n *metabolites *X*_*1*_*, X*_*2*_*, ..., X*_*i*_*, ..., X*_*n*_, and for each metabolite, a time series consisting of *N *time points *t*_*1*_*, t*_*2*_*, ..., t*_*k*_*, ..., t*_*N *_has been observed. If we can measure or deduce the slope *S*_*i*_*(t*_*k*_*) *for each metabolite at each time point, we can reformulate the system as *n *sets



Thus, for the purpose of parameter estimation, the original system of *n *coupled differential equations can be analyzed in the form of *n *× *N *uncoupled algebraic equations [4,9].

The uncoupling step renders the estimation of slopes a crucial step. If the data are more or less noise-free, simple linear interpolation, splines [10-12], B-splines [13], or the so-called three-point method [14] are effective. If the data are noisy, it is useful to smooth them, because the noise tends to be magnified in the slopes. Established smoothing methods again include splines, as well as different types of filters, such as the Whittaker filter (see [15] for a review), collocation methods [16], and artificial neural networks [17,18]. In order to keep our illustration of the AR method as clean as possible, we assume that true slopes are available and elaborate on issues of experimental noise in the *Discussion*.

### Alternating regression

**Figure 1:**
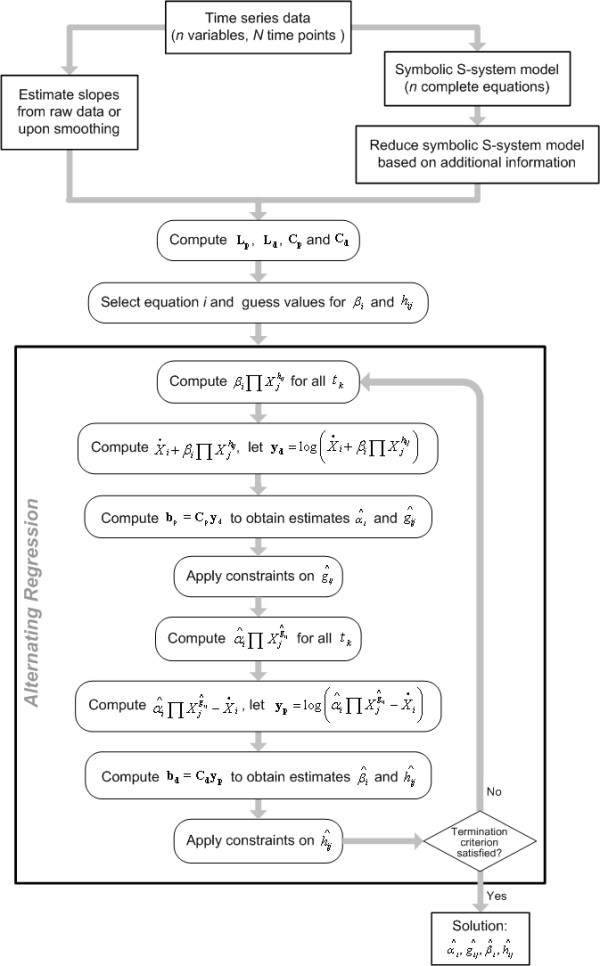
Logistic flow of parameter estimation by alternating regression.

The decoupling of the system of differential equations allows us to estimate the S-system parameters *α*_*i*_*, g*_*ij*_*, β*_*i*_, and *h*_*ij*_* (i, j = 1,2,...,n) *one equation at a time, using slopes and concentration values of each metabolite at time points *t*_*k*_. The proposed method called *alternating regression *(AR) has been used in other contexts such as spectrum reconstruction and robust redundancy analysis [19,20], but, to the best of our knowledge, not for the purpose of parameter estimation from time series. The overall flow of the method is shown in Figure 1. Adapted to our task of S-system estimation, AR works by cycling between two phases of multiple linear regression. The first phase begins with guesses of all parameter values of the degradation term in a given equation and uses these to solve for the parameters of the corresponding production term. The second phase takes these estimates to improve the prior parameter guesses or estimates in the degradation term. The phases are iterated until a solution is found or AR is terminated for other reasons.

In pure parameter estimation tasks, the structure of the underlying network is known, so that it is also known which of the S-system parameters are zero and which of the kinetic orders are positive or negative. Thus, the search space is minimal for the problem. Nonetheless, the same method of parameter estimation can in principle also be used for structure identification. In this case, the estimation is executed with an S-system where no parameter is *a priori *set to zero and all parameters have to be estimated. As an intermediate task, it is possible that only some of the structure is known. This information can again be used to reduce the search space. If it is known, for instance, that variable *X*_*j *_does not affect the production or degradation of *X*_*i*_, the corresponding parameter value *g*_*ij *_or *h*_*ij *_is set equal to zero, or *X*_*j *_is taken out of the regression. One can thus reduce the regression task either by constraining the values of some *g*'s or *h*'s throughout the AR or by selecting a subset of regressors at the beginning, *i.e*., by taking some variables out of the regression. Similarly, if a kinetic order is known to represent an inhibiting (activating) effect, its range of possible values can be restricted to negative (positive) numbers. This constraining of kinetic orders, while not essential, typically improves the speed of the search. It is imaginable that a kinetic order is constrained too tightly. In this case, the solution is likely to show the kinetic order at the boundary, which is subsequently relaxed.

To estimate the parameters of the *i*^th ^differential equation, the steps of the AR algorithm are as follows:

{1} Let **L**_**p **_denote an *(n+1) *× *N *matrix of logarithms of regressors *X*_*i*_, defined as



**L**_**p **_is used in the first phase of AR to determine the parameter values of the production term. Additional information on the system, if it is available, reduces the width of **L**_**p**_. For instance, if *X*_*2*_and *X*_*4*_do not affect the production of *X*_*1*_in a four variable system, Eq. (3) reduces to



Analogous to **L**_**p**_, let **L**_**d **_denote the *(n+1) *× *N *matrix of regressors used in the second phase of AR to determine the parameter values of the degradation term. **L**_**p **_and **L**_**d **_are the same when the variables used in two phases of AR are identical.

{2} Compute the matrices

**C**_**p **_= **(L**_**p**_^**T**^**L**_**p**_**)**^**-1**^**L**_**p**_^**T **^    (5)

**C**_**d **_= **(L**_**d**_^**T**^**L**_**d**_**)**^**-1**^**L**_**d**_^**T **^    (6)

which are invariant throughout the iterative process.

{3} Select values for *β*_*i *_and *h*_*ij *_in accordance with experience about S-system parameters (*cf*. [4]: Ch. 5) and make use of any available information constraining some or all *h*_*ij*_.

{4} For all *t*_*k*_, *k *= 1, 2, ..., *N*, compute , using values *X*_*j*_*(t*_*k*_*) *from the observed or smoothed time series measurements.

{5} Compute the *N*-dimensional vector  (*k *= 1, 2, ..., *N*) containing transformed "observations" on the degradation term. *Note*: It is possible to compute **y**_**d **_for all *n *traces simultaneously so that **Y**_**d **_becomes an *n *× *N *matrix with columns **y**_**d**_.

{6} Based on the multiple linear regression model

**y**_**d **_= **L**_**p**_**b**_**p **_+ **ε**_**p **_    (7)

estimate the regression coefficient vector **b**_**p**_= [, , *j *= 1, 2,..., *n*]' by regression over the *N *time points. In other words, this step leads to an estimation of parameters in sets of equations of the type

. Specifically, compute **b**_**p **_as

**b**_**p **_= **(L**_**p**_^**T**^**L**_**p**_**)**^**-1**^**L**_**p**_^**T**^**y**_**d **_= **C**_**p**_**y**_**d **_    (8)

according to Eqs. (3–5).

{7} Constrain some or all , if outside information on the model suggests it.

{8} Using the observed values of *X*_*j*_*(t*_*k*_*)*, compute  for all *t*_*k*_, *k *= 1, 2, ..., *N*.

{9} Compute the *N*-dimensional vector  containing the transformed "observations" associated with the production term.

{10} Based on the multiple linear regression model

**y**_**p **_= **L**_**d**_**b**_**d **_+ **ε**_**d **_    (9)

and in analogy to step {6}, estimate the regression coefficient vector **b**_**d **_= [, , *j *= 1, 2,..., *n*]' by regression over the *N *time points as

**b**_**d **_= **C**_**d**_**y**_**p **_    (10)

{11} Constrain some or all , if outside information on the model suggests it.

{12} Iterate Steps {4} - {11} until a solution is found or some termination criterion is satisfied.

At each phase of AR, lack-of-fit criteria are estimated and used for monitoring the iterative process and to define termination conditions. In this paper we use the sum of squared *y*-errors (*SSE*_*d *_and *SSE*_*p*_) as optimization criteria for the two regression phases, *i.e*. we compute



where  = **L **× **b**, **L **equals **L**_**p **_or **L**_**d**_, and **b **is the solution vector **b**_**p **_or **b**_**d**_, estimated through regression and modified by constraints reflecting structural information. We use the logarithm of *SSE *because it is superior in illustrating small changes in the residual error.

It is known that collinearity may affect the efficiency of multivariate linear regressions. We therefore also implemented methods of principal component regression (PCR), partial least squares regression (PLSR) and ridge regression [21]. For the cases analyzed here, these methods did not provide additional benefit.

## Results and discussion

For illustration purposes, we use a didactic system with four variables that is representative of a small biochemical network [5]. A numerical implementation with typical parameters is



**Figure 2:**
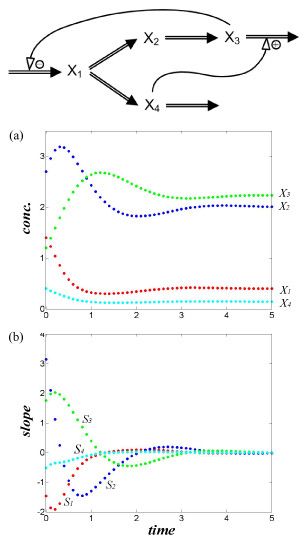
**Test system with four dependent variables**. (a) time courses computed with initial values in Eq. (12) (use dataset 1 in Table S1); (b) corresponding dynamics of slopes. Typical units might be concentrations (*e.g*., in mM) plotted against time (*e.g*., in minutes), but the example could as well run on an hourly scale and with variables of a different nature.

The system is first used to create artificial datasets that differ in their initial conditions (Table S1 of *Additional file*1). In a biological setting, these may mimic different stimulus-response experiments on the same system. For example, they could represent different nutrient conditions in a growth experiment. Figure 2 shows the branched pathway, along with a selection of time course data (dataset 1) and slopes.

In order not to confuse the features of AR with possible effects of experimental noise, we use true metabolite concentrations and slopes; we compute the latter directly from Eq. (12) at each time point. We initially assume that we have observations at 50 time points, but discuss cases with fewer points and with noise later.

### Performance of AR

Given the time series data of *X*_*i *_and *S*_*i*_ at every time point *t*_*k*_, the AR algorithm is performed for each metabolite, one at a time. Figure S1 summarizes various patterns of convergence observed. Generally we can classify the convergence patterns into four types: 1) convergence to the true value; 2) convergence to an incorrect value; 3) no convergence; typically the value of *α*_*i *_(or *β*_*i*_) continuously increases while all *g*_*ij *_(or *h*_*ij*_) gradually approach zero, while in some other cases *g*_*ij *_and the corresponding *h*_*ij *_increase (or decrease) in a parallel manner; 4) termination during AR, due to some of the observations **y**_**d **_(or **y**_**p**_) taking on complex values.

As is to be expected, the speed of convergence depends on the initial guesses, the variables used as regressors, the constraints, and the data set. After a few initial iterations, the approach of the true value is usually, though not always, strictly monotonic. In some cases, the error initially decreases rapidly and subsequently enters a phase of slower decrease. It is also possible that convergence is non-monotonic, that the algorithm converges to a different point in the search space, or that it does not converge at all. Convergence to the wrong solution and situations of no convergence are particularly interesting. In the case of no convergence, the solution arrives at unreasonable parameter values that grow without bound; this case is very easy to detect and discard. By contrast, the search may lead to a solution with wrong parameter values, but a satisfactory residual error. Thus, the algorithm produces a wrong, but objectively good solution. It is close to impossible with *any *algorithm to guard against this problem, unless one can exclude wrong solutions based on the resulting parameter values themselves. This is actually greatly facilitated with S-systems because all parameters have a clearly defined meaning in terms of both their sign and magnitude, which may help spot unrealistic solutions with small residual error.

Reasons for AR not to converge are sometimes easily explained, but sometimes obscure. For instance, the slope-minus-degradation or -production expressions in steps {5} and {9} of the algorithm may become negative, thereby disallowing the necessary logarithmic transformation. As a consequence, the regression terminates. If this happens, it usually happens during the first or the second iteration, and the problem is easily solved when the initial *β* or *α* is increased. In other cases, AR converges for one dataset, but not for another, even for the same model. This sometimes happens if datasets have low information content, for instance, if the dynamics of a variable is affected by a relatively large number of variables, but the observed time course is essentially flat or simple monotonic. In this case, convergence is obtained if one adjusts the constraints on some of the parameter values or selects a different set of regressors (see below). Of importance is that each iteration consists essentially of two linear regressions so that the process is fast. Thus, even the need to explore alternative settings is computationally cheap and provides for an effective solution to the convergence problem.

### Patterns of convergence

The speed and pattern of convergence depend on a combination of several features, including initial guesses for all parameters and the datasets. Overall, these patterns are very complicated and elude crisp analytical evaluations. This is not surprising, because even well-established algorithms like the Newton method can have basins of attraction that are fractal in nature (*e.g*., [22]). A detailed description of some of these issues, along with a number of intriguing color plates describing well over one million ARs, is presented in Additional file 1.

#### Effect of initial parameter guesses

**Figure 3:**
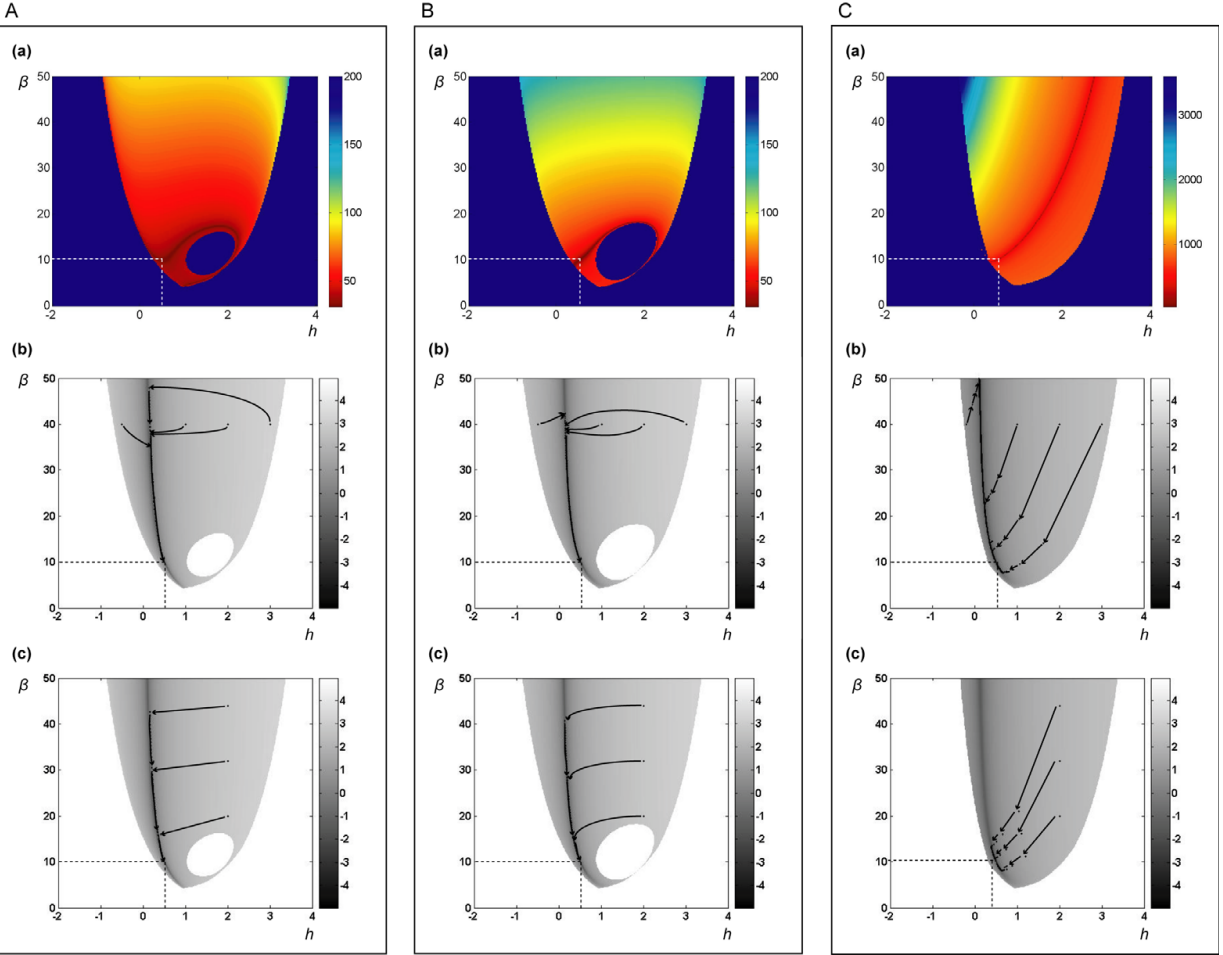
**Summary of convergence patterns of AR**. Panel A: all variables are initially used as regressors and constraints are imposed afterwards; Panel B: regression with the "union" of variables of both terms; Panel C: only those variables that are known to appear in the production or degradation term, respectively, are used as regressors. Row (a): speed of convergence; the color bars represent the numbers of iterations needed to converge to the optimum solution; Rows (b) and (c): 2D view of the error surface superimposed with convergence trajectories with different initial values of *β* and *h*; the color bars represent the value of *log(SSE)*. The intersections of dotted lines indicate the optimum values of parameters *β* and *h*.



Figure 3 combines results from several sets of initial guesses of *β*_*i *_and *h*_*ij *_(the results of the second phase of AR are not shown, but are analogous). The data for this illustration consist of observations on the first variable of datasets 4, 5 and 6 (see Table S1 in the Additional file1). These are processed simultaneously as three sets of algebraic equations at 50 time points. Thus, the parameters *α*_*1*_, *g*_*13*_, *β*_*1*_, and *h*_*11*_of the equation

are to be estimated. As a first example, we initiate AR with all variables (*X*_*1*_, ..., *X*_*4*_) as regressors, but constrain the kinetic orders *g*_*11*_, *g*_*12*_, and *g*_*14*_to be zero after the first phase of the regression, and the kinetic orders *h*_*12*_, *h*_*13*_, and *h*_*14*_after the second phase, in accordance with the known network structure.

Figure 3A(a) shows the "heat map" of the convergence, where the x- and y-axes represent the initial guesses of *h*_*11*_and *β*_*1*_, respectively, and the color bar represents the number of iterations needed for convergence. Since we use noise-free data, the residual error should approaches 0, which corresponds to -∞ in logarithmic coordinates. We use -7 instead as one of the termination criteria, which corresponds to a result very close to the true value, but allows for issues of machine precision and numerical inaccuracies. Once this error level is reached, AR stops and the number of iterations is recorded as a measure for the speed of convergence. The unusual shape of a "martini with olive" is due to the following. The deep blue outside area indicates an inadmissible domain, where the initial parameter guess causes one or more of the terms  in step {5} to become negative, so that the logarithm, **y**_**d**_, becomes a complex number and the regression cannot continue. The line separating admissible and inadmissible domains is thus not smooth but shows the envelope of several pieces of power-law functions where the *β*-term is smaller than the (negative) slope at some time point. The "olive" inside the glass is also inadmissible. In this case, the chosen initial value causes the term  in step {9} to become negative, so that **y**_**p **_becomes complex and AR terminates during the second phase. This type of termination usually, though not always, happens during the first iteration. In order to prevent it, one may *a priori *require that



for every *t*_*k*_, such that the logarithm is always defined. This is possible through the choice of a sufficiently large value for the initial guess of *β*. The magnitude of *β* should be reasonable, however, because excessive values tend to slow down convergence. As a matter of practically, one may start with a value of 5 or 10 and double it if condition (14) is violated.

#### Use of different variables as regressors

Panel A in Figure 3 shows results where we initially use all variables as regressors, but constrain their kinetic orders to zero after each iteration, if they are known to be zero. As alternatives, Panels B and C show results of using different variable combinations as regressors under otherwise identical conditions. In Panel B, both phases of AR use all variables as regressors that appear in either the production or the degradation term of the equation. In Panel C we make full use of our knowledge of the pathway structure and include in each term only the truly involved variables. Interestingly, this choice of regressors has a significant effect on convergence.

Compared with the case in of Figure 3A(a), the speed of convergence is slower in Figure 3B(a) and much slower in Figure 3C(a), even though this represents the "best-informed" scenario. The time needed to generate the graphs in Figures 3A(a), 3B(a), and 3C(a) for all shown 60,000 initial values is 72, 106, and 1,212 minutes, respectively. Thus, if we suppose that roughly half of the start points are inadmissible and require no iteration time, the average convergence time in Figure 3A(a) is 0.144 seconds, whereas it is 0.212 seconds in Figure 3B(a) and 2.424 seconds in Figure 3C(a). The pattern of convergence is affected by the datasets used. As another example, Figure S2 shows results of regressions with dataset 5.

#### Error surface

Rows (b) and (c) in Figure 3 Panels A, B, and C show heat maps of *log(SSE)*, where darker dots indicate smaller errors. The true minimal value of *log(SSE) *for our noise-free data is -∞, but for illustration propose, we plot it only to -5. Pseudo-3-D graphs of the error surface are shown in Figure S3 with views from two angles.

#### Convergence trajectories

Paths toward the correct solution may be visualized by plotting and superimposing the solution at every regression step onto the corresponding heat maps, with arrowheads indicating the direction of each trajectory (Figures 3A(b,c), 3B(b,c), and 3C(b,c)). For the first set of illustrations, four different initial values of *h*_*11*_are chosen, while the value of *β*_*1*_is always 40. For the second set of illustrations, four different initial values of *β*_*1*_are chosen, while the value of *h*_*11*_is always 2. Interestingly, independent of the start values, only two iterations are needed to reach a point very close to the valley of the error surface where the true solution is located. After the dramatic initial jump, all solutions follow essentially the same trajectory with small steps toward the true solution. We can also link the observations of Figure 3A(b) and 3A(c) to the result in 3A(a). For the same β_1_, a start point in the right part the graph causes AR to jump to a more distant location on the trajectory, thus requiring more iterations to converge to the true solution.

It might be possible to speed up convergence in the flat part of the error surface, for instance by using history-based modeling based on conjugated gradients or partial least squares regression [21]. These options have not been analyzed.

#### Accuracy and speed of solution

The previous sections focused on the first equation of the S-system model in Eq. (12) and Figure 2. We used the AR algorithm in the same manner to estimate all other parameters. Again, three sets of regressors were used for every variable. For simplicity of discussion, we describe the results from using dataset 1 of Table S1, always using as initial guesses *β*_*i *_= 15 and *h*_*ij *_= 1. The results are listed in Tables 1, S2 and S3.

**Table 1:** Estimated parameter values of the S-system model of the pathway in Figure 2 using *log(SSE) *< -7 as termination criterion. ^a ^Regressor: A: all variables used as regressors and subsequently constrained; B: use of "union" variables as regressors (see *Text*); C: fully informed selection of regressors (see *Text*). ^b ^time (secs) needed to converge to the solution with *log(SSE) *< -7. ^c ^Convergence results according to AR algorithm: *: convergence to the true solution; **: convergence to different solution; ***: no convergence. ^d ^time after running 1,000,000 iterations. See Eq. (12) for optimal parameter values and the *Additional file *for further comments.

	Regressor^a^	*α*_*i*_	*g*_*i*1_	*g*_*i*2_	*g*_*i*3_	*g*_*i*4_	*β*_*i*_	*h*_*i*1_	*h*_*i*2_	*h*_*i*3_	*h*_*i*4_	*log(SSE)*	Time(sec)^b^	Note^c^
X_1_	A	12.00	0.00	0.00	-0.80	-0.00	10.00	0.50	-0.00	0.00	0.00	-6.84	0.58	*
	B	12.03	-0.00	0	-0.80	0	10.04	0.50	0	0.00	0	-7.00	2.39	*
	C	12.00	0	0	-0.80	0	9.99	0.50	0	0	0	-6.95	0.17	*

X_2_	A	44.50	-0.00	-0.02	-0.04	0.11	31.48	0.03	0.14	0.05	-0.13	0.51	1071.58^d^	**
	B	8.01	0.50	0.00	0	0	3.01	-0.00	0.75	0	0	-7.00	0.97	*
	C	8.01	0.50	0	0	0	3.01	0	0.75	0	0	-7.00	69.05	*

X_3_	A	3.00	0.00	0.75	-0.00	-0.00	5.00	-0.00	0.00	0.50	0.20	-9.44	0.03	*
	B	7.29	0	0.37	-0.00	-0.00	8.76	0	-0.00	0.19	0.04	-4.04	1117.14^d^	**
	C	2.98	0	0.75	0	0	5.00	0	0	0.51	0.20	-7.01	0.50	*

X_4_	A	96.80	0.01	0.01	-0.00	0.00	100.00	-0.00	-0.01	0.00	0.02	-3.83	4.59	***
	B	98.29	0.06	0	0	0.00	100.00	-0.00	0	0	0.01	-5.85	341.94	***
	C	2.016	0.50	0	0	0	5.99	0	0	0	0.80	-6.97	84.91	*

For every variable, at least one of the three choices of regressors leads to convergence to the correct solution. Convergence is comparably fast, even if we require a very high accuracy for termination (*log*(*SSE*) < -20) (see Table S2). If we relax the accuracy to *log*(*SSE*) < -7 or *log*(*SSE*) < -4, the solution is still very good, but the solution time is noticeably decreased (Tables 1 and S3). However, the false-positive rate increases slightly for *log*(*SSE*) < -4. As a compromise, we use *log*(*SSE*) < -7 as termination criterion for the remainder of this paper.

Interestingly, the speed of convergence is fastest for the strategy "A" of using all variables as regressors; however, the failure rate in this case is also the highest. In contrast, the slowest speed of convergence is obtained for the correct regressors ("C"), where AR always converges to the right solution. The regressor set "B" is between "A" and "C" in terms of speed and ability to yield the correct optimum. For cases that don't converge to the right solution one easily adapts the AR algorithm by choosing different start values, slightly modifying constraints, or choosing different regressors in addition to the three types used above. The probability of finding the correct solution is increased if different datasets are available for sequential or simultaneous estimation. The same was observed for other estimation methods (*e.g*., [5]).

### Structure identification

The previous sections demonstrated parameter estimation for a system with known structure. Similar to this task is the identification of the unknown structure of a pathway from time series data, if one uses S-systems as the modeling framework [5]. The only difference is that very few or no parameters at all can *a priori *be set to zero or constrained to the positive or negative half of the search space. A totally uninformed AR search of this type often leads to no convergence. However, since each AR is fast, it is feasible to execute many different searches, in which some of the parameters are allowed to float, while others are set equal to zero.

Table S6 shows the results of exhausting all combinations of constraints to determine those that yield convergence. The total time for this exhaustive search is just over one hour. This is furthermore reduced if some *a priori *information is available. As an alternative to an exhaustive search, one may obtain constraining information from a prior linearization of the system dynamics [23]. This method does not identify parameter values per se, but provides very strong clues on which variables are likely to be involved in a given equation and which not. In the example tested, this method provided an over 90% correct classification of the relevant variables in each equation (see Table S7). Using this inference information, the total time was reduced to 53 minutes.

Finally, it is possible to sort parameter combinations by their empirical likelihood of inclusion in an equation [24]). For instance, a metabolite usually affects its own degradation but usually has no effect on its own production. Thus, a reasonable start is the parsimonious model  with *g*_*ii *_= 0 and *h*_*ii *_> 0. In subsequent runs, free-floating variables (parameters) are added, one at a time. This strategy reduced the total time from one hour to under 3 minutes (see Table S8). As illustration, and for a second, independent example, we used the strategy of Veflingstad *et al*. [23] to determine the regulatory structure and parameter values of a gene regulatory network model [25] that has become a benchmark in the field. Kikuchi and collaborators [6] identified the structure of this model by using a genetic algorithm acting directly on the five differential equation of the model. Using a cluster of 1,040 CPUs, the solution required about 70 hours. We generated time series data from the model, using 0.5 as initial concentration for all five variables. The solution time needed for exhausting all constraint combinations for all variables and an error tolerance of *log(SSE) *= -7 was 81.2 min on a single PC. Interestingly, the false-positive rate in this case was higher in this system as compared to the example above. The time needed for the hierarchical strategy proposed by Marino and Voit [24] was 6.38 mins. The parameter values of metabolites *X*_*1*_, *X*_*2*_, *X*_*4*_, and *X*_*5*_ were found correctly, but the parameters associated with *X*_*3*_ were not all identified, even though the error satisfied our termination criterion (*log(SSE) *< -7), indicating that a different solution with essentially zero-error exists in this equation. This result interestingly echoes the result based on linearization, as proposed by Veflingstad *et al*. [23]. The reason is probably that *X*_*2*_ contributes to both the production term and the degradation term of *X*_*3*_ with the same kinetic order (-1) and that the time course is not very informative. Also similar to Veflingstad's results, when we used different initial concentrations to perturb *X*_*2*_ and *X*_*3*_ more strongly, AR yielded the correct solution.

## Conclusion

Biological system models are usually nonlinear. This renders the estimation of parameter values a difficult problem. S-systems are no exception, but we have shown here that their regular structure offers possibilities for restructuring the estimation problem that are uniquely beneficial. Specifically, the combination of the previously described method of decoupling with the alternating regression technique proposed here dramatically reduces estimation time. Since the AR algorithm essentially consists of iterative linear regressions, it is extremely fast. This makes it feasible to explore alternative settings or initial guesses in cases where a particular initiation fails to lead to convergence.

Methods of parameter estimation, and the closely related task of structure identification, naturally suffer from combinatorial explosion, which is associated with the number of equations and the much faster increasing number of possible interactions between variables, which show up as parameters in the equations. The proposed method of decoupling behaves much better in this respect than most others (*cf*. [5,24]). In practical applications, the increase in the number of combinations is in most cases vastly less than theoretically possible, because the average connectivity of a biological network is relatively small (<<*O*(*n*^2^); *e.g*., [26]).

The patterns of convergence are at this point not well understood. Some issues were discussed in the *Results *section and others are detailed in Additional file 1. From these numerical analyses it is clear that convergence depends in a very complicated fashion on the dataset, the constraints, the choice of regressors, and the structure and parameter values of the system. Given that even the convergence features of the Newton algorithm are not fully understood [22], it is unlikely that simple theorems will reveal the convergence patterns of AR in a general manner.

The speed of convergence is also affected by the starting guesses, the choice of regressors, the constraints imposed, and the data set. From our analyses so far it seems that if initially more regressors are used than actually needed, and if they are secondarily constrained, AR converges the fastest. However, a loosely constrained selection of regressors also has a higher chance of convergence to a wrong solution or never to converge. This is especially an issue if the time series are not very informative; for instance, if the system is only slightly perturbed from its steady state. By contrast, when fewer regressors are used, the speed of convergence is slower, but the chance of reaching the optimal solution is increased. A possible explanation of this phenomenon is that more regressors offer more degrees of freedom in each regression, which results in more leeway but also in an increased chance for failure. If AR does not converge, choosing different datasets, using different regressors, or slightly relaxing or tightening the constraints often yields convergence to the correct solution. Most importantly, in all cases of convergence the solution is obtained very quickly in comparison to other methods that attempt to estimate parameters directly via nonlinear regression on the differential equations.

At this stage we have deduced optimized solutions for each metabolite separately. In other words, we have not accounted for constraints among equations, such as stoichiometric precursor-product or branch point relationships. Also, it seems that similar methods should be efficacious for the estimation of Generalized Mass Action systems [4]. These issues will be the subject of further study. We have also assumed that the data are error-free. This assumption was made to identify advantages and failures of the AR algorithm in a fashion as unobstructed as possible. Also, as we typically smooth raw data before estimating parameter values, the analysis of noisy data seems to depend more on the quality of smoothing than on AR itself. The same is the case for data that do not stem from S-system models, where the quality of the estimation is driven by the accuracy of the S-system representation. Future studies will elucidate how sensitive to experimental error the algorithm is.

Like any other estimation algorithm, AR is not a panacea. However, our studies so far provide strong indication that this algorithm is much faster than nonlinear algorithms that one can afford to test quite a number of false starts and explore multiple combinations of initial guesses.

## Competing interests

The author(s) declare that they have no competing interests.

## Supplementary Material

Additional File 1 Additional file of the manuscript Click here for file
