# Supplementary material for: Parameter estimation in biochemical systems models with alternating regression
Source: Theor Biol Med Model. 2006 Jul 19;3:25. doi: 10.1186/1742-4682-3-25 (PMC1586003; doi:10.1186/1742-4682-3-25)
Supplement: Additional File 1 — Additional file of the manuscript [file 1742-4682-3-25-S1.pdf]

Additional file

to *Parameter Estimation in Biochemical Systems Models with Alternating Regression*

by I-Chun Chou, Harald Martens, and Eberhard O. Voit

1. Further Documentation of Patterns of Convergence.

As discussed in the *Text*, convergence depends on a number of factors, such as the types of regressors used. Following are tables and figures cited in the *Text*.

Table S1. Sets of initial concentrations used for the creation of artificial datasets.

Dataset	$X_1(t_0)$	$X_2(t_0)$	$X_3(t_0)$	$X_4(t_0)$
1	1.4	2.7	1.2	0.4
2	0.4	2.0	4.5	0.1
3	0.2	0.3	2.2	0.01
4	2.0	2.0	2.2	0.1
5	1.4	1	0.2	3.0
6	4.0	1.0	3.0	4.0

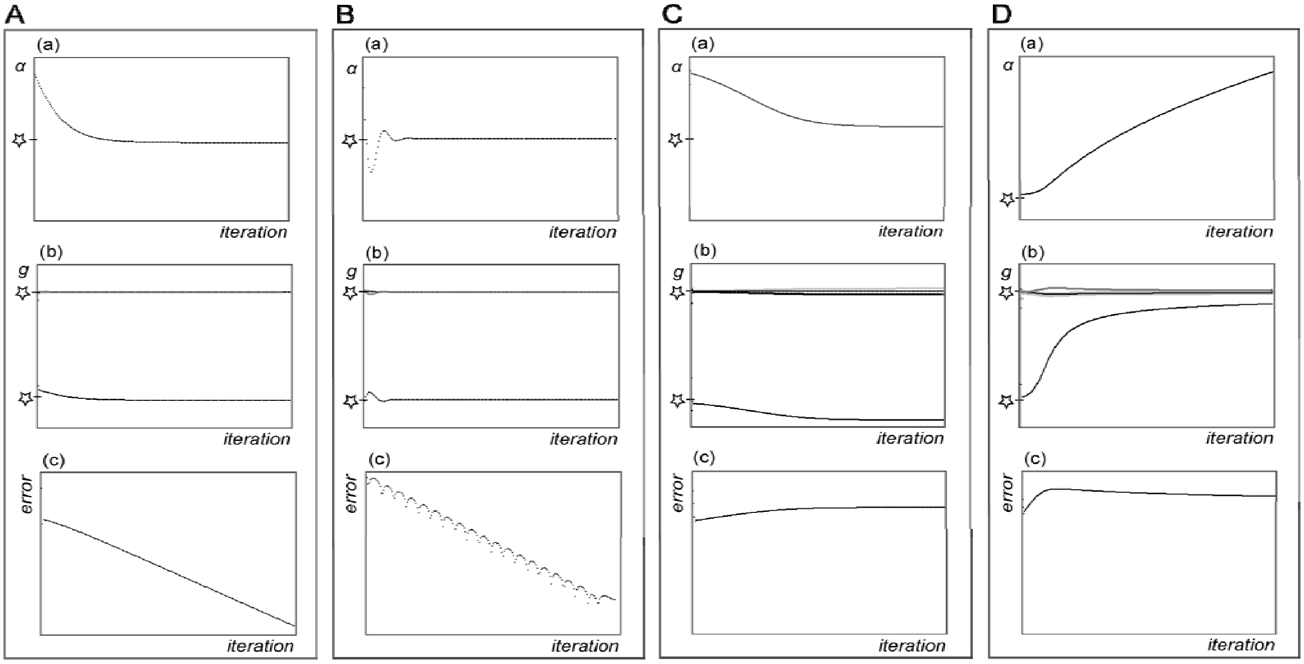

Figure S1. Generic patterns of convergence of AR. Panel A: monotonic convergence to the true value; Panel B: non-monotonic convergence to the true value; Panel C: convergence to a different value; Panel D: no convergence. Row (a): rate constant α; Row (b): kinetic order g; Row (c): log of residual error. The asterisk represents the true value of α or g. See *Text* for detailed description.

Patterns of convergence (Figure S1). Given the time series data of X_i and S_i at every time point t_k, the AR algorithm is performed for each metabolite, one at a time. Figure S1 summarizes various patterns of convergence observed. The abscissa indicates the iteration number and the ordinate the value of one of the parameter estimates or the error. As example, we show changes in the estimates of α_i, g_{ij}, and $log(SSE(\alpha_i, g_{ij}))$, given an initial guess of β_i and h_{ij}. Estimated values of β_i and h_{ij} and of $log(SSE(\beta_i, h_{ij}))$ in the second phase are not shown but exhibit similar patterns. The asterisk represents the true value of α_i or g_{ij}. Generally we can classify the convergence patterns into four types: 1) convergence to the true value; 2) convergence to an incorrect value; 3) no convergence; typically the value of α_i (or β_i) continuously increases while all g_{ij} (or h_{ij}) gradually approach zero, while in some other cases g_{ij} and the corresponding h_{ij} increase (or decrease) in a parallel manner; 4) termination during AR, due to some of the observations $\mathbf{y_d}$ (or $\mathbf{y_p}$) taking on complex values.

Panel A of Figure S1 represents the case of "convergence to the true solution" with gradually decreasing error. As is to be expected, the speed of convergence depends on the initial guesses, the variables used as regressors, the constraints, and the data set. After a few initial iterations, the approach of the true value is usually, though not always, strictly monotonic. In some cases, the error initially decreases rapidly and subsequently enters a phase of slower decrease. Panel B also shows cases of convergence, but with non-monotonic progress. Panel C represents cases of convergence to a different point in the search space. As in Panels A and B, the search converges, sometimes to a solution close to the true solution, but at other times to a distinctly different solution, yet with an error that is acceptable while not quite as good as in A and B. Panel D represents the cases of no convergence; without intervention, the system does not recover from this trend.

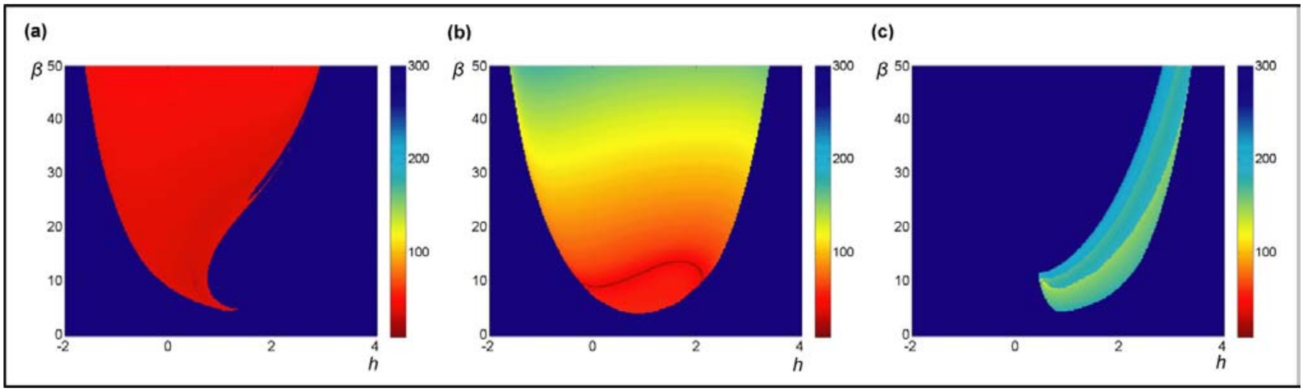

Figure S2. Convergence of AR for data set 5, using (a) all variables with secondary constraints; (b) "union" variables; and (c) fully informed variable selection (see *Text* for details).

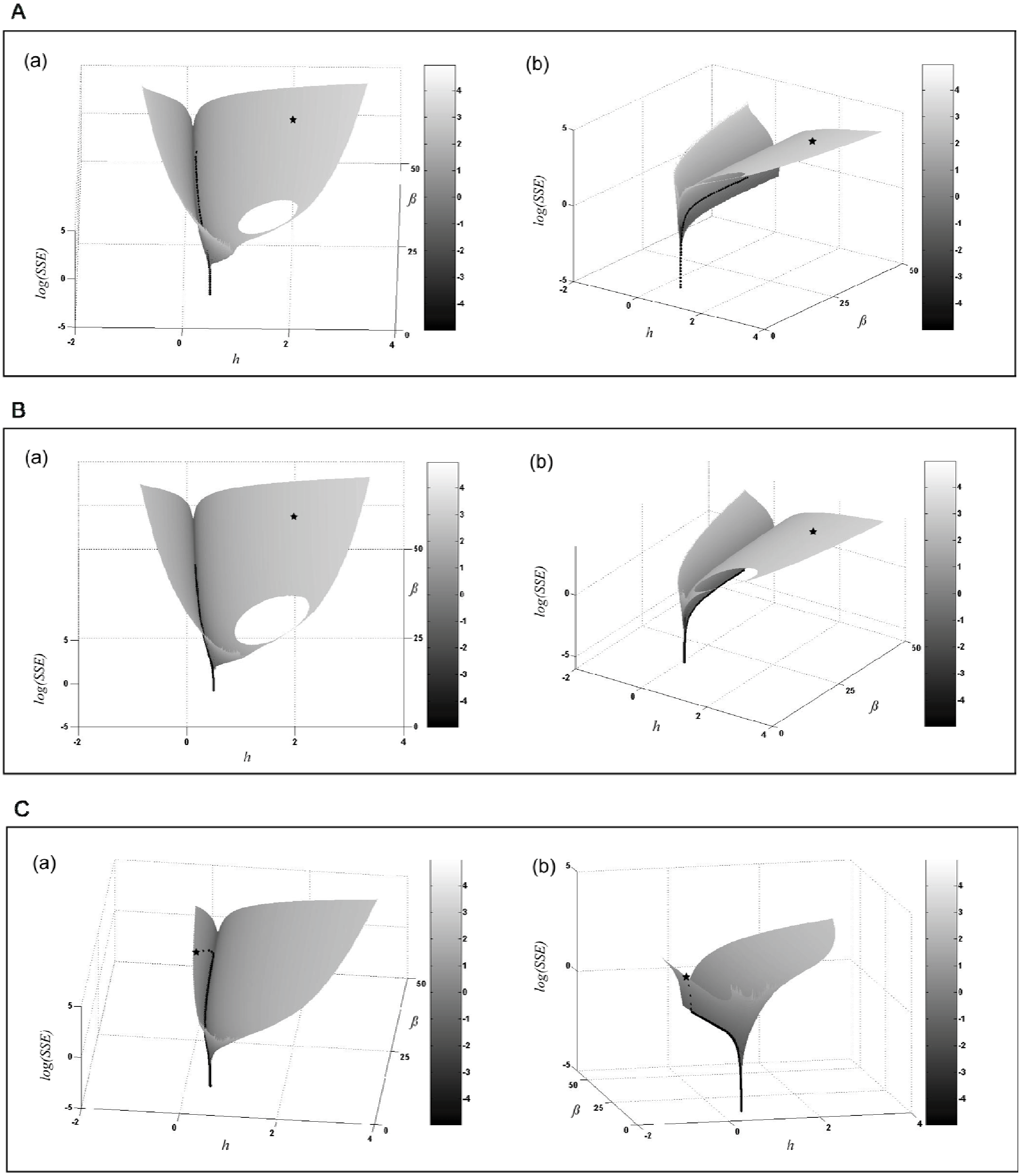

Figure S3. Pseudo-3D graph of the error surface for a convergence trajectory starting from (β, h) = (40, 2) (indicated as *). The graphs in Panels A, B, and C correspond to the graphs in Figure 3 (Panels A, B, and C), respectively. Columns (a) and (b) show views from two angles. A(a, b) show the same convergence trajectory (initiated at β_I=40, h_{II}=2; indicated by an asterisk) in a pseudo-3-dimensional plot. In just one iteration, the trajectory is close to—though not exactly in—the valley of the error surface.

Accuracy and speed of solution. The following tables (S2-S3) correspond to Table 1 in the *Text*, but use different error thresholds. In all cases, convergence depends on a number of factors, such as the selection of data sets. If we choose data sets 1, 2, and 5, for example, then AR converges quickly to the right solution for metabolite X_4. Slightly modifying constraints after each phase of AR is another strategy to improve the likelihood of convergence. For example, in the case of metabolite X_2, we could relax the true constraints from [g_{21} 0 0 0] [0 h_{22} 0 0] to more generalized combinations like [g_{21} 0 0 g_{24}] [0 h_{22} h_{23} 0], [g_{21} 0 g_{23} g_{24}] [0 h_{22} 0 0], or [g_{21} g_{22} 0 g_{24}] [0 h_{22} 0 0], where 0 indicates exclusion of the corresponding variable. In all these cases AR converges quickly to the right solution. In other words, even we don't constrain some parameters to zero that should truly be 0, the AR algorithm automatically forces them to approach zero. It appears that the relaxing of constraints gives the AR algorithm more space to find the optimal solution. Using different combinations of regressors can also help. Again, in the case of metabolite X_4, if we use metabolite X_1 and X_3 to fit the model in the first phase of AR and then use metabolites X_1, X_3, and X_4 to fit the model in the second phase of AR, the algorithm successfully converges to the correct solution. This trial and error approach may appear somewhat *ad hoc*, but exploring several combinations in troublesome cases is still considerably faster than any competing algorithm that we aware of.

Table S2. Estimated parameter values of the S-system model of the pathway in Figure 2 using *log(SSE)* < -20 as termination criterion.

	Regressor[a]	α_i	g_{i1}	g_{i2}	g_{i3}	g_{i4}	β_i	h_{i1}	h_{i2}	h_{i3}	h_{i4}	*log(SSE)*	Time(secs)[b]	Note[c]
X_1	A	12.00	0.00	0.00	-0.80	-0.00	10.00	0.50	-0.00	0.00	0.00	-19.18	0.97	*
	B	12.00	-0.00	0	-0.80	0	10.00	0.50	0	0.00	0	-20.00	5.48	*
	C	12.00	0	0	-0.80	0	10.00	0.50	0	0	0	-19.94	270.97	*
X_2	A	44.50	-0.00	-0.02	-0.04	0.11	31.48	0.04	0.14	0.05	-0.13	0.51	1062.83[d]	**
	B	8.00	0.50	0.00	0	0	3.00	-0.00	0.75	0	0	-20.01	1.95	*
	C	8.00	0.50	0	0	0	3.00	0	0.75	0	0	-20.00	103.39	*
X_3	A	3.00	0.00	0.75	-0.00	-0.00	5.00	-0.00	0.00	0.50	0.20	-19.79	0.05	*
	B	7.29	0	0.37	-0.00	-0.00	8.76	0	-0.00	0.19	0.04	-4.04	1111.63[d]	**
	C	3.00	0	0.75	0	0	5.00	0	0	0.50	0.20	-20.00	589.97	*
X_4	A	96.80	0.01	0.01	-0.00	0.00	100.00	-0.00	-0.01	0.00	0.02	-3.83	3.50	***
	B	98.29	0.01	0	0	0.00	100	-0.00	0	0	0.01	-5.85	340.34	***
	C	2.00	0.50	0	0	0	6.00	0	0	0	0.80	-19.97	289.09	*

[a] Regressor: A: all variables used as regressors and subsequently constrained; B: use of "union" variables as regressors (see *Text*); C: fully informed selection of regressors (see *Text*). [b] time (secs) needed to converge to the solution with *log(SSE)*<-20. [c] *: convergence to the true solution; **: convergence to different solution; ***: no convergence. [d] time after running 1,000,000 iterations.

Table S3. Estimated parameter values of the S-system model of the pathway in Figure 2 using *log(SSE)* < -4 as termination criterion.

	Regressor[a]	α_i	g_{i1}	g_{i2}	g_{i3}	g_{i4}	β_i	h_{i1}	h_{i2}	h_{i3}	h_{i4}	*log(SSE)*	Time(secs)[b]	Note[c]
	A	12.04	0.00	0.00	-0.79	-0.00	10.07	0.50	-0.00	0.00	0.00	-3.84	0.44	*
X_1	B	13.81	-0.00	0	-0.60	0	12.16	0.38	0	0.00	0	-4.00	0.94	*
	C	12.29	0	0	-0.83	0	10.08	0.51	0	0	0	-3.92	0.06	*
	A	44.50	-0.00	-0.02	-0.04	0.11	31.48	0.03	0.14	0.05	-0.13	0.51	1073.05[d]	**
X_2	B	8.47	0.46	0.00	0	0	3.42	-0.00	0.69	0	0	-4.00	0.58	*
	C	8.46	0.46	0	0	0	3.42	0	0.69	0	0	-4.00	59.91	*
	A	3.00	0.00	0.75	-0.00	-0.00	5.00	-0.00	0.00	0.50	0.20	-9.44	0.06	*
X_3	B	3.81	0	0.63	-0.00	-0.00	5.60	0	-0.00	0.39	0.13	-4.65	0.03	*
	C	2.80	0	0.83	0	0	5.56	0	0	0.63	0.31	-4.01	0.20	*
	A	96.80	0.01	0.01	-0.00	0.00	100.00	-0.00	-0.00	0.00	0.02	-3.83	4.52	***
X_4	B	10.08	0.06	0	0	0.00	11.98	-0.00	0	0	0.12	-3.98	1.72	*[e]
	C	2.24	0.40	0	0	0	5.60	0	0	0	0.66	-3.97	29.42	*

[a] Regressor: A: all variables used as regressors and subsequently constrained; B: use of "union" variables as regressors (see *Text*); C: fully informed selection of regressors (see *Text*). [b] time (secs) needed to converge to the solution with *log(SSE)* < -4. [c] *: convergence to the true solution; **: convergence to different solution; ***: no convergence. [d] time after running 1,000,000 iterations. [e] false positive.

Density of sampling points. Instead of using time series with 50 sampling points, we applied AR to data sets with only 10 points, consisting of the same starting and ending time, but larger time intervals. The results (Table S4) demonstrate that the density of time points in this case does not affect the efficacy of AR if the data are noise free. In addition to increasing the intervals between data points, we also reduced the time series from 50 observations to the first 25 points. The results (Table S5) show that AR still converges in most cases to the true solution.

Noisy data and data from non-S-system models. As is typical with demonstrations of new algorithms in this field, it is beneficial at first to concentrate on error-free data in order to investigate how well the algorithm works under ideal conditions. In cases of noise-corrupted (artificial or real) data, we typically smooth our data with methods like the three-point method, some smoother like the Whitaker filter, or an artificial neural network (see discussion in [1]). If the raw data are smoothed before application of the proposed (or other) algorithm(s), the question of the effects of noise in truth become questions of the power, reliability, and efficiency of the chosen smoother. Similarly, if the data represent a model that is not optimally modeled with an S-system, the issue is not so much the proposed search algorithm as the quality of the S-system representation. We will analyze these issues elsewhere in greater detail.

Table S4. Estimated parameter values of the S-system model of the pathway in Figure 2 using *log(SSE)* < -7 as termination criterion with 10 sampling points.

	Regressor[a]	α_i	g_{i1}	g_{i2}	g_{i3}	g_{i4}	β_i	h_{i1}	h_{i2}	h_{i3}	h_{i4}	*log(SSE)*	Time(secs)[b]	Note[c]
	A	11.99	0.00	0.00	-0.80	-0.00	9.99	0.50	-0.00	0.00	0.00	-4.66	0.45	*
X_1	B	12.07	0.00	0	-0.79	0	10.10	0.49	0	0.00	0	-7.00	1.80	*
	C	12.07	0	0	-0.79	0	10.10	0.49	0	0	0	-6.99	14.72	*
	A	50.56	-0.20	-0.06	-0.22	0.25	27.54	0.098	0.11	0.26	-0.30	0.72	544.03	**
X_2	B	8.02	0.50	-0.00	0	0	3.02	-0.00	5	0	0	-7.00	0.76	*
	C	8.02	0.50	0	0	0	3.02	0	0.75	0	0	-6.98	27.00	*
	A	3.00	-0.00	0.75	-0.00	0.00	5.00	-0.00	-0.00	0.50	0.20	-12.84	0.02	*
X_3	B	3.07	0	0.74	-0.00	-0.00	5.06	0	-0.00	0.49	0.19	-6.81	0.49	*
	C	3.04	0	0.75	0	0	5.08	0	0	0.50	0.20	-7.00	0.20	*
	A	96.11	0.02	0.00	0.00	0.00	100.00	-0.00	-0.00	-0.00	0.03	-3.41	2.86	***
X_4	B	98.28	0.01	0	0	0.00	100.00	-0.00	0	0	0.01	-6.40	87.09	***
	C	2.01	0.49	0	0	0	5.97	0	0	0	0.79	-6.98	34.00	*

[a] Regressor: A: all variables used as regressors and subsequently constrained; B: use of "union" variables as regressors (see *Text*); C: fully informed selection of regressors (see *Text*). [b] time (secs) needed to converge to the solution with *log(SSE)* < -7. [c] *: convergence to the true solution; **: convergence to different solution; ***: no convergence. [d] time after running 1,000,000 iterations.

Table S5. Estimated parameter values of the S-system model of the pathway in Figure 2 using *log(SSE)* < -7 as termination criterion the first 25 points.

	Regressor[a]	α_i	g_{i1}	g_{i2}	g_{i3}	g_{i4}	β_i	h_{i1}	h_{i2}	h_{i3}	h_{i4}	log(SSE)	Time(secs)[b]	Note[c]
	A	100.05	-0.00	-0.01	-0.05	0.02	96.72	0.05	0.01	-0.00	-0.02	-1.44	0.55	***
X_1	B	12.03	-0.00	0	-0.79	0	10.05	0.50	0	0.00	0	-6.93	3.73	*
	C	12.03	0	0	-0.79	0	10.05	0.50	0	0	0	-6.92	21.53	*
	A	73.18	4.92	-4.89	6.94	0.80	1.27	0.53	0.47	1.28	-0.37	1.96	0.03	***
X_2	B	8.0	0.50	0.00	0	0	3.01	-0.00	0.75	0	0	-7.01	0.72	*
	C	8.01	0.50	0	0	0	3.01	0	0.75	0	0	-7.00	36.49	*
	A	3.00	0.00	0.75	-0.00	-0.00	5.00	-0.00	0.00	0.50	0.20	-7.06	0.08	*
X_3	B	3.02	0	0.75	-0.00	-0.00	5.00	0	-0.00	0.49	0.120	-6.3	0.19	*
	C	3.03	0	0.74	0	0	5.00	0	0	0.49	0.19	-7.00	30.53	*
	A	97.33	0.01	0.00	-0.00	0.00	100.00	-0.00	-0.00	0.00	0.02	-3.87	6.38	***
X_4	B	98.49	0.00	0	0	0.00	100.00	-0.00	0	0	0.01	-6.19	196.98	***
	C	2.01	0.50	0	0	0	5.97	0	0	0	0.79	-6.94	71.56	*

[a] Regressor: A: all variables used as regressors and subsequently constrained; B: use of "union" variables as regressors (see *Text*); C: fully informed selection of regressors (see *Text*). [b] time (secs) needed to converge to the solution with *log(SSE)* < -7. [c] *: convergence to the true solution; **: convergence to different solution; ***: no convergence. [d] time after running 1,000,000 iterations.

2. Further Documentation Regarding Structure Identification.

Table S6. Constraints on kinetic orders leading to AR convergence. Termination criterion is *log(SSE)* < -7.

	Production constraint	Degradation constraints	α_i	g_{i1}	g_{i2}	g_{i3}	g_{i4}	β_i	h_{i1}	h_{i2}	h_{i3}	h_{i4}	Time*
X_1	$[0\ 0\ g_{13}\ 0]$	$[h_{11}\ 0\ 0\ 0]$	12.00	0.00	0.00	-0.80	0.00	10.00	0.50	0.00	0.00	0.00	
	$[0\ 0\ g_{13}\ 0]$	$[h_{11}\ h_{12}\ 0\ 0]$	12.00	0.00	0.00	-0.80	0.00	10.00	0.50	0.00	0.00	0.00	
	$[0\ g_{12}\ g_{13}\ 0]$	$[h_{11}\ 0\ 0\ 0]$	12.00	0.00	0.00	-0.80	0.00	10.00	0.50	0.00	0.00	0.00	
	$[g_{11}\ 0\ g_{13}\ 0]$	$[h_{11}\ 0\ 0\ 0]$	12.02	0.00	-0.00	-0.80	-0.00	10.02	0.50	0.00	0.00	0.00	20.82
X_2	$[g_{21}\ 0\ 0\ g_{24}]$	$[0\ h_{22}\ h_{23}\ 0]$	8.02	0.50	0.00	0.00	0.00	3.00	-0.00	0.75	0.00	0.00	
	$[g_{21}\ 0\ g_{23}\ g_{24}]$	$[0\ h_{22}\ 0\ 0]$	8.04	0.50	-0.00	-0.00	0.00	3.01	0.00	0.75	-0.00	-0.00	
	$[g_{21}\ g_{22}\ 0\ g_{24}]$	$[0\ h_{22}\ 0\ 0]$	7.97	0.50	0.00	-0.00	-0.00	2.99	0.00	0.75	-0.00	-0.00	8.50
X_3	$[0\ g_{32}\ 0\ 0]$	$[0\ 0\ h_{33}\ h_{34}]$	3.00	0.00	0.75	-0.00	-0.00	5.00	-0.00	0.00	0.5	0.2	
	$[0\ g_{32}\ g_{33}\ 0]$	$[0\ 0\ h_{33}\ h_{34}]$	3.00	0.00	0.75	-0.00	-0.00	5.02	0.00	-0.00	0.50	0.20	9.21
X_4	$[g_{41}\ 0\ 0\ 0]$	$[0\ h_{42}\ 0\ h_{44}]$	2.00	0.50	-0.00	-0.00	-0.00	6.00	0.00	-0.00	0.00	0.80	
	$[g_{41}\ 0\ 0\ 0]$	$[0\ h_{42}\ h_{43}\ h_{44}]$	2.02	0.49	0.00	0.00	-0.00	6.02	-0.00	-0.00	0.00	0.80	
	$[g_{41}\ 0\ 0\ g_{44}]$	$[0\ 0\ 0\ h_{44}]$	2.06	0.49	-0.00	0.00	0.01	6.08	-0.00	0.00	-0.00	0.80	
	$[g_{41}\ 0\ g_{43}\ 0]$	$[0\ h_{42}\ 0\ h_{44}]$	2.03	0.49	0.00	-0.00	-0.00	6.03	-0.00	-0.00	-0.00	0.79	
	$[g_{41}\ g_{42}\ 0\ 0]$	$[0\ 0\ h_{43}\ h_{44}]$	2.01	0.50	0.00	0.00	-0.00	6.00	-0.00	0.00	0.00	0.80	
	$[g_{41}\ g_{42}\ g_{43}\ 0]$	$[0\ 0\ 0\ h_{44}]$	2.02	0.49	0.00	-0.00	-0.00	6.01	-0.00	0.00	-0.00	0.79	30.60

* Time (mins) needed for testing all 256 combinations of zero and non-zero values of kinetic orders in each equation.

Table S7. Collective inference of the gene network based on results from all linearization, according to Veflingstad *et al.* (2004).

	X_1	X_2	X_3	X_4
X_1	**- (100 %)**	**0 (100 %)**	**- (83 %)**	**0 (83 %)**
X_2	**+ (100 %)**	**- (67 %)**	**0 (100 %)**	**0 (83 %)**
X_3	**0 (100 %)**	**+ (83 %)**	**- (83 %)**	0 (67 %)
X_4	**+ (100 %)**	**0 (100 %)**	**0 (100 %)**	**- (83 %)**

A plus sing implies a positive influence, a minus sign implies a negative influence, and a zero implies no influence. Bold entries denote correctly identified interactions and numbers in parentheses give the fraction of models that suggest positive identification.

The savings with this method in the given example are actually only modest (about 20%). Among the possible reasons are that the method does not allow distinction between effects mediated through the α-term from those mediated through the β-term and that the interaction between X_3 and X_4 (represented by g_{34} and h_{34}) is actually not identified correctly, even though Veflingstad's method gives it 66.7% support. Forcing g_{24} and h_{24} to be zero (which is predicted to be the case with 83% likelihood) leads to no convergence.

Table S8. First constraint found leading to AR convergence, starting from the most parsimonious constraint. Termination criterion is *log(SSE)* < -7.

	Production constraint	Degradation constraints	α_i	g_{i1}	g_{i2}	g_{i3}	g_{i4}	β_i	h_{i1}	h_{i2}	h_{i3}	h_{i4}	Time*
X_1	$[0\ 0\ g_{13}\ 0]^a$	$[h_{11}\ 0\ 0\ 0]^a$	12.00	0.00	0.00	-0.80	0.00	10.00	0.50	0.00	0.00	0.00	0.02
X_2	$[g_{21}\ g_{22}\ 0\ g_{24}]^b$	$[0\ h_{22}\ 0\ 0]^b$	7.97	0.50	0.00	-0.00	-0.00	2.99	0.00	0.75	-0.00	-0.00	0.95
X_3	$[0\ g_{32}\ 0\ 0]^c$	$[0\ 0\ h_{33}\ h_{34}]^c$	3.00	0.00	0.75	-0.00	-0.00	5.00	-0.00	0.00	0.5	0.2	0.49
X_4	$[g_{41}\ 0\ 0\ g_{44}]^d$	$[0\ 0\ 0\ h_{44}]^d$	2.06	0.49	-0.00	0.00	0.01	6.08	-0.00	0.00	-0.00	0.80	0.86

* Time (mins) needed for testing all 256 combinations of zero and non-zero values of kinetic orders in each equation. [a] In the 4th place of the combination matrix 1; [b] In the 31th place of the combination matrix 2; [c] In the 20th place of the combination matrix 3; [d] In the 11th place of the combination matrix 4.

3. Numerical Characterization of AR's Basin of Attraction for Different Datasets.

Any analytical characterization of the convergence of a nonlinear search algorithm for dynamical models is a very demanding task. Even for the Newton algorithm, which has been used and analyzed by generations of researchers in mathematics, computer science, and various application fields, convergence can be extremely complex and essentially impossible to predict. As an example, Epureanu and Greenside [2], as well as numerous original papers and websites, review the basins of attraction for this algorithm, which even in really simple cases of algebraic functions can consist of very complicated fractals. The same is true for every other nonlinear search algorithm, including Levenberg-Marquardt, genetic algorithms, and simulated annealing, where it is close to impossible to predict with reliability whether a search will succeed in finding the true solution.

Given this complexity and the long history of the Newton algorithm and other search algorithms, it is not likely that we will be able to develop crisp and general theorems characterizing the convergence behavior of our new algorithm. Indeed, it seems not possible with present

mathematical means to characterize the convergence features of our proposed algorithm in generality. As the next best alternative, we have therefore chosen to pursue the topic with a comprehensive computational analysis (comprising with over 1,000,000 alternative regressions) of two examples [1, 3], which have become something like unofficial case studies for comparisons of algorithms in the field. In addition to the discussions in the *Text*, we describe here the effects of using different datasets from the same system, which are characterized by different initial values of the dependent variables. We have recently shown [4] with “time-dependent sensitivities” how initial values affect the dynamics of trajectories. The analysis here illuminates a related issue, but from a different angle.

In order to demonstrate the effects of initial conditions on convergence, we investigated in great detail dataset 1 of system in Figure 2, with initial conditions $X_1(t_0)$ = Int1 = 1.4, $X_2(t_0)$ = Int2 = 2.7, $X_3(t_0)$ = Int3 = 1.2, and $X_4(t_0)$ = Int4 = 0.4. To allow for a two-dimensional representation, we fixed Int3 and Int4 and changed Int1 and Int2 (essentially exhaustively) in different combinations. Figures S4, S6 and S8 represent the 2-D “dataset convergence maps” of using all variables as regressors, “union” variables as regressors, and variables that are known to appear in each term as regressors, respectively (as described in *Text*). Each map consists of about 160,000 alternating regression analyses, where each dot represents a dataset. The color of the dot codes for the number of iterations needed to converge to the right solution, starting with the same initial guesses of β_i and h_{ij} that we used as example in the paper. The color scales are the same in three figures.

The main result is that the “convergence maps” in Figures S4, S6 and S8 are very complicated. They do not seem to be fractal as in the Newton case, but in some sense even more complicated by not revealing obvious patterns. Striped areas represent domains in the space where the logarithm of some slope minus one power-law term is not defined, as described in detail in the paper. In a nutshell, using a dataset from within these areas, and again starting with the initial guess of β_i and h_{ij}, the expressions in steps {5} and {9} of the algorithm become negative, thereby disallowing the necessary logarithmic transformation. Shaded areas represent no-convergence areas. When using datasets from within these areas, the value of α_i (or β_i) typically increases continuously and without bound while some or all g_{ij} (or h_{ij}) gradually approach zero; in some other cases g_{ij} and the corresponding h_{ij} increase (or decrease) in a parallel manner. These situations seem to indicate low information content of the dataset.

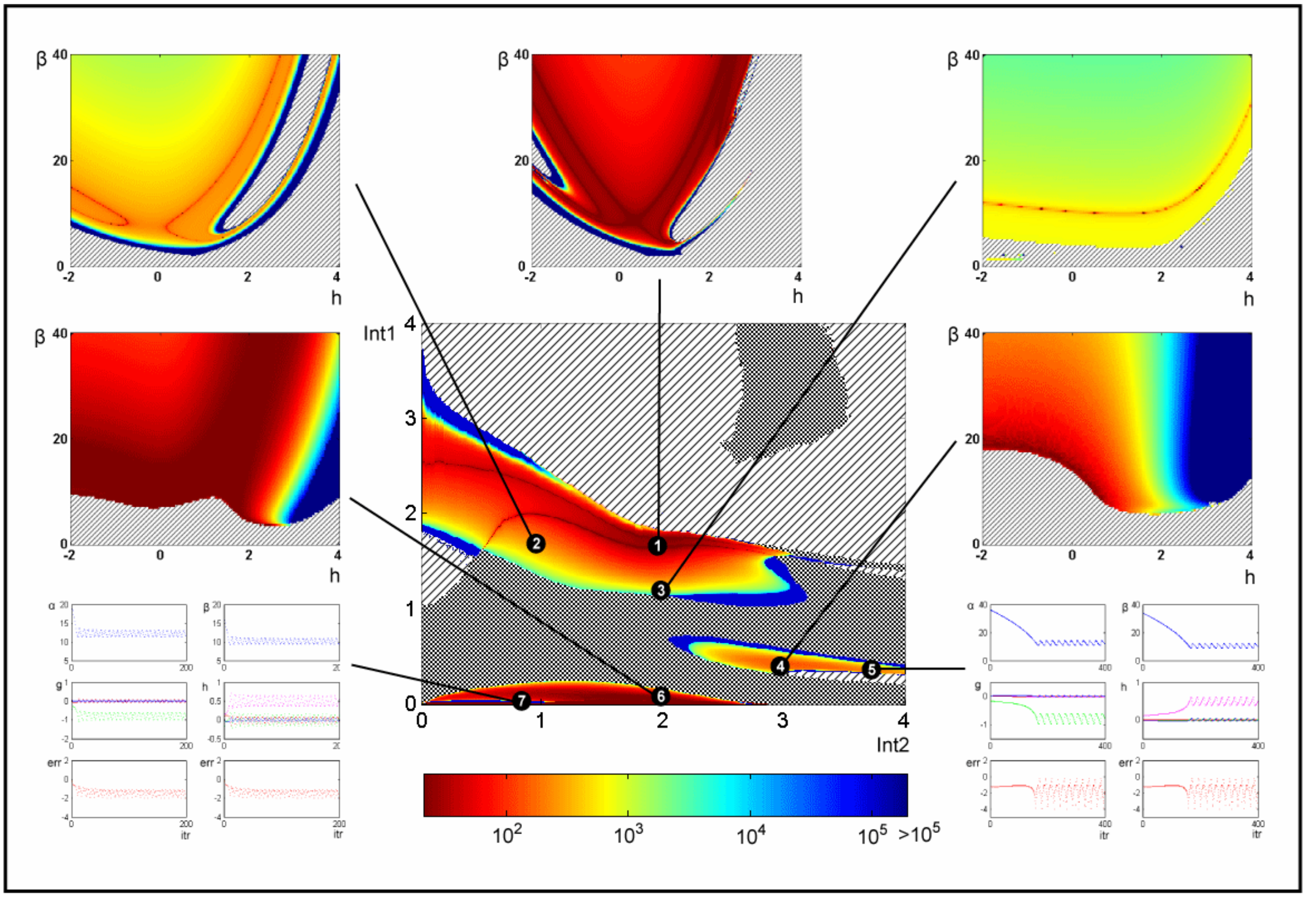

Figure S4. Use of all variables as regressors (see *Text* for general explanations).

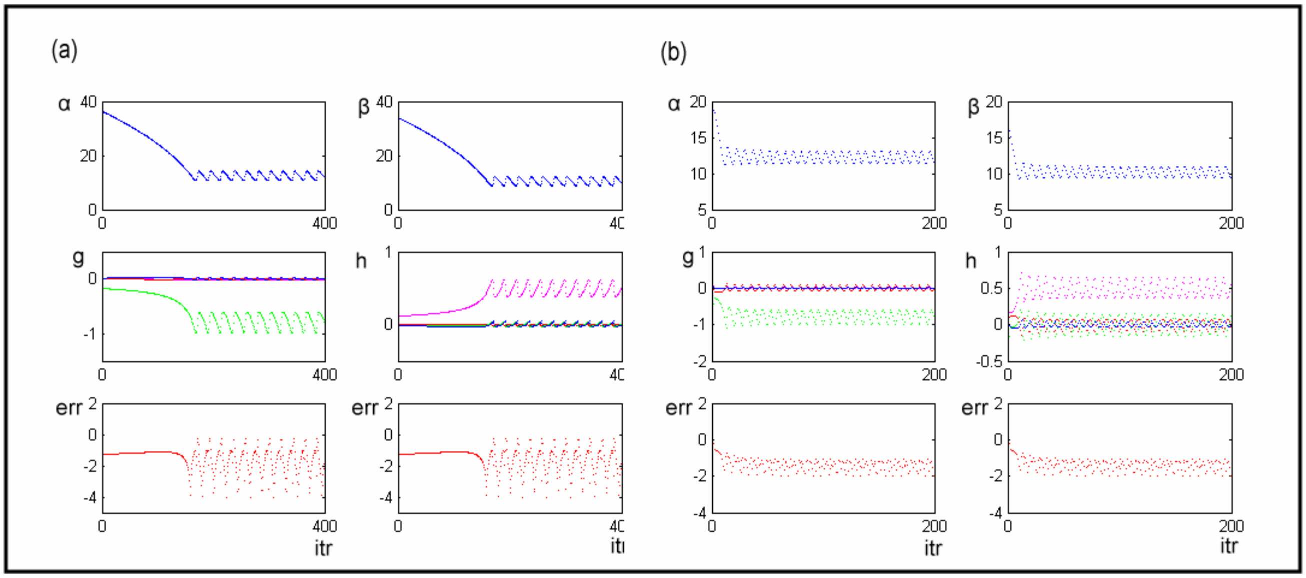

Figure S5. (a) Close-up of Figure S4 ❺; (b) Close-up of Figure S4 ❼.

The satellite figures around the central plots in Figures S4, S6, and S8 represent the convergence maps of particular datasets. These plots show the effects of changing the start guesses (β_i and h_{ij}) used in the alternating regression, given the particular dataset indicated by a number. Figure S5 represents close-ups of figures identified as ❺ and ❼ in Figure S4. In these cases, the parameter values are oscillating near the true solutions. Such two-cycle oscillations are not unusual in iterative searches. The changes in parameter values within the non-convergence (shaded) areas are similar in Figures S4, S6, and S8. One representative example is shown in Figure S6 ❼.

Figure S4 has the largest "problem" areas. However, outside these areas convergence is very fast. Intriguingly, the problem areas are substantially reduced in size when one uses fewer variables as regressors (*i.e.*, if the degrees of freedom are decreased). For instance, Figure S8 does not even have a "no-convergence" area. Interestingly, and not yet fully explained, the convergence speed in these cases is usually much slower than in Figures S4 and S6.

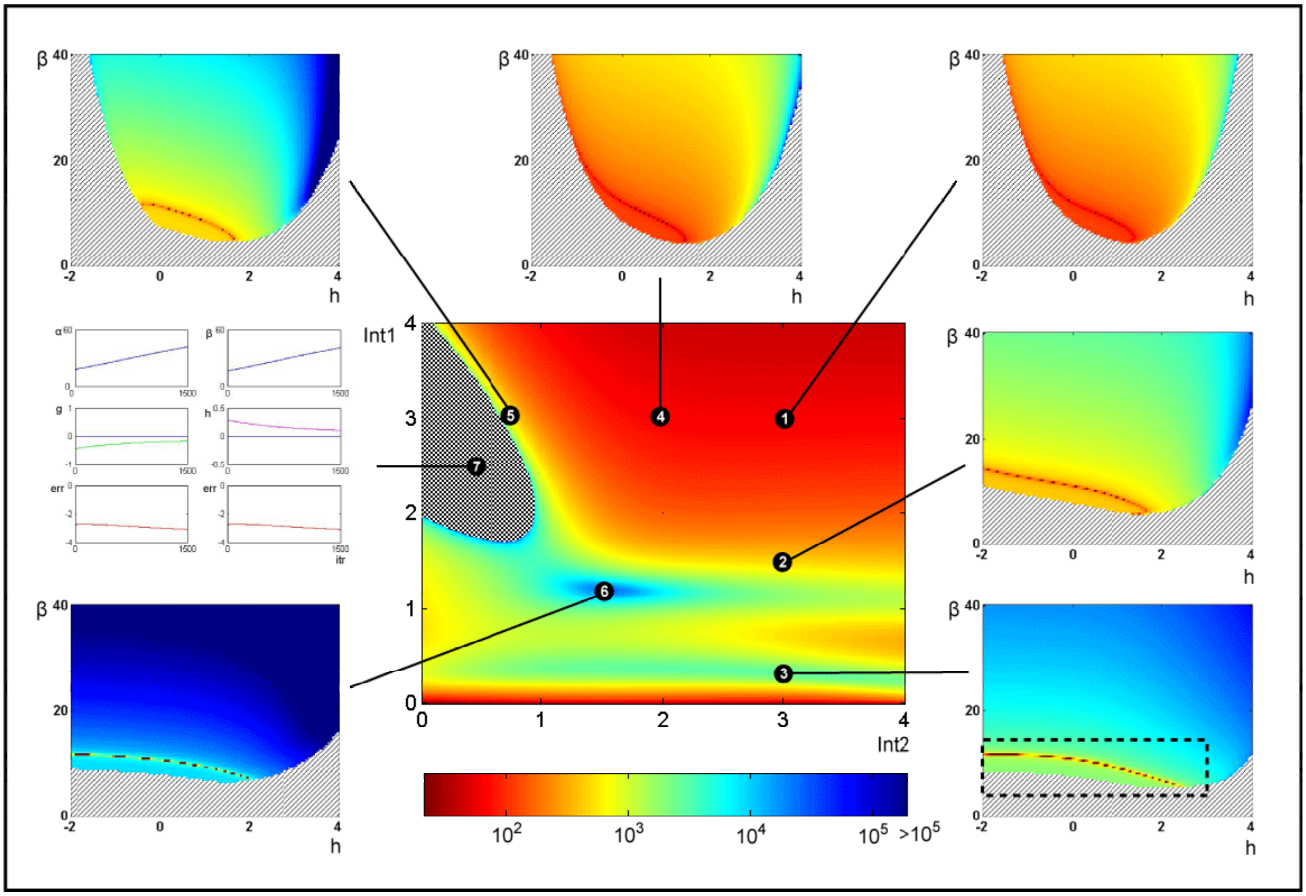

Figure S6. Use of the "union" of variables as regressors (see *Text* for general explanations).

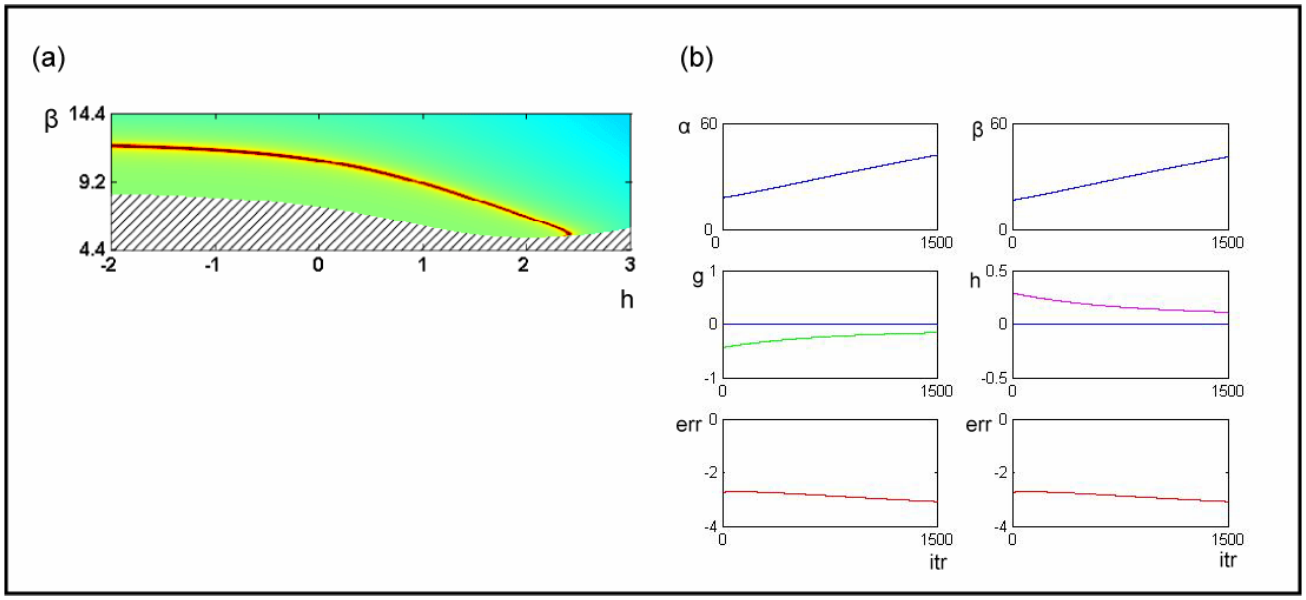

Figure S7. (a) Close-up of Figure S6 ❸; (b) Close-up of Figure S6 ❼.

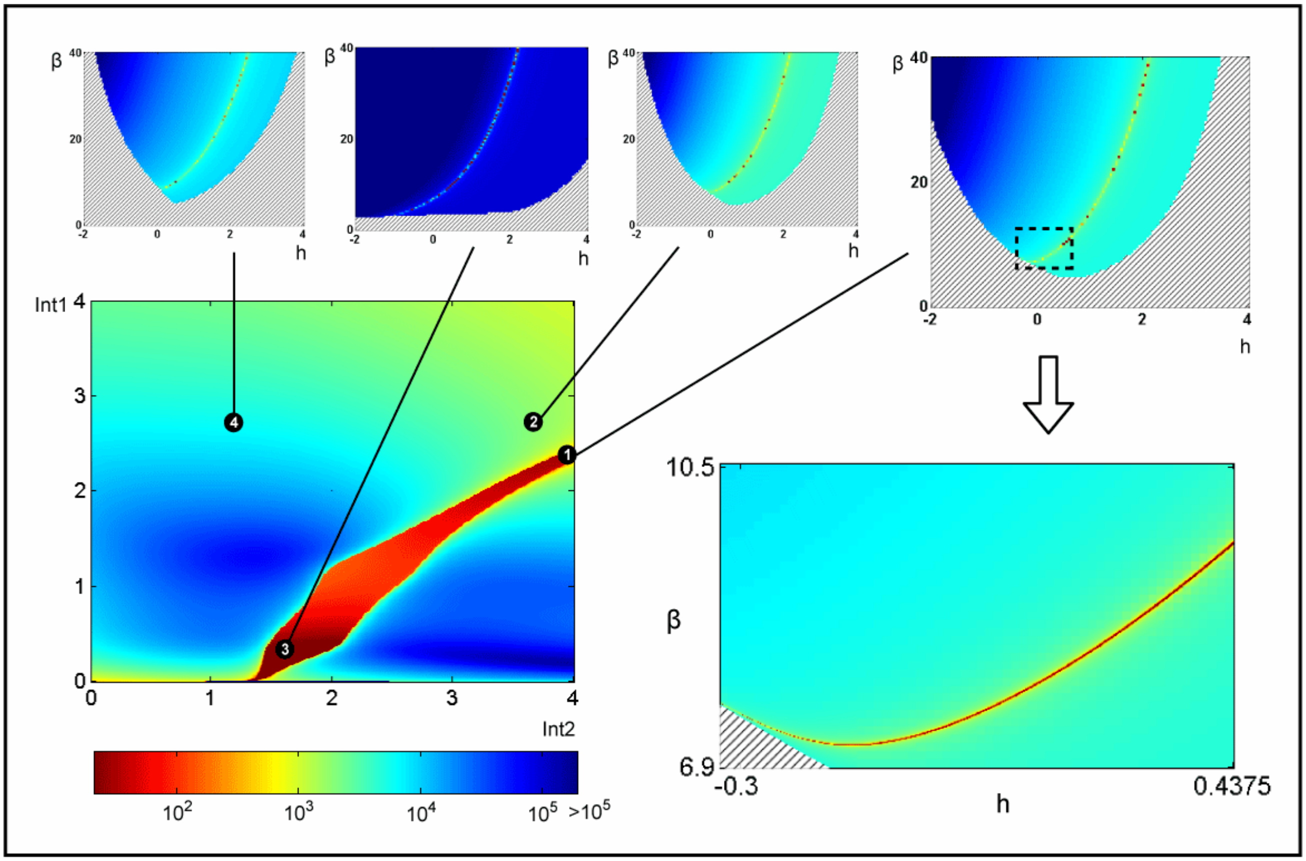

Figure S8. Use of variables that are known to appear in each term as regressors (see *Text* for general explanations).

The graphs in Figures S4, S6, and S8 provide strong indication that it will be very difficult to determine the convergence areas analytically, especially when more variables then necessary are

used as regressors. Even for some points very close to each other, their convergence properties could be entirely different (see Figure S7 (a)): One point may lead to convergence to the right solution, while "neighbors" may not converge or lead to negative arguments in the logarithmic transformations in steps {5} or {9}. In contrast to highly symmetric fractal pictures often associated with the Newton method, the basin of attraction here is complicated and does not suggest an intuitive pattern.

So far we only varied the values of Int1 and Int2, in order to facilitate a graphical representation. The question then becomes how changing Int1, Int2, Int3, and Int4 simultaneously would affect convergence. Because of the complexity of the situation we can only show select results of how the initial conditions affect the number of iterations needed when we use as regressors those variables that are known to appear in each term.

Figure S9 shows some results of partial least square regression (PLS), elucidating this situation. In this case, Int1, Int2 and Int3 are statistically significant (striped areas) in predicting the number of iterations needed. Int1 and Int2 contribute negatively to convergence speed, while Int3 significantly increases the number of iterations needed; changing Int4 has no significant effect. These results have to be considered with caution, because they are highly dependent not only on the initial values, but also on the error threshold and other factors. A more comprehensive study, including all contributing factors will be needed.

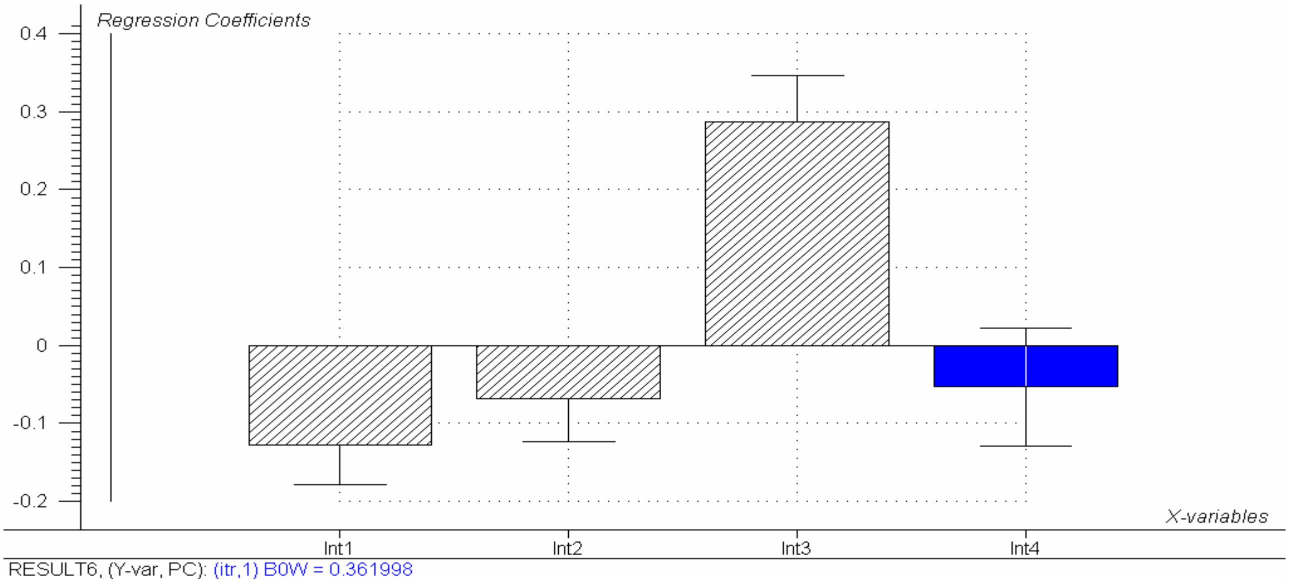

Figure S9. Results of the partial least squares (PLS) analysis, indicating the influence of initial values of variables X_1-X_4 on convergence speed. *Unscrambler*® software was used.

Finally, we designed a multiple-level full-factorial experiment to identify which design variable (initial condition) influences response (number of iterations needed) significantly and which not. Table S9 shows that seven effects are found to be significant, four of them are confounded interactions. Again, these results have to be considered with caution. A more comprehensive study, including all contributing factors will be needed.

Table S9. Results of an ANOVA characterizing the influence of initial values of variables X_1-X_4 on convergence speed. *Unscrambler*® software was used.

	SS	DF	MS	F-ratio	p-value
Summary					
Model	4.820e+12	170	2.836e+10	6.444	0.0000
Error	4.950e+12	1125	4.400e+09		
Adjusted Total	9.771e+12	1295	7.545e+09		
Variable					
Int1	5.840e+10	5	1.168e+10	2.654	0.0215
Int2	3.924e+11	5	7.847e+10	17.834	0.0000
Int3	1.854e+12	5	3.708e+11	84.260	0.0000
Int4	3.248e+10	5	6.497e+09	1.476	0.1947
(Int1)(Int2)	6.184e+11	25	2.474e+10	5.622	0.0000
(Int1)(Int3)	2.746e+11	25	1.098e+10	2.496	0.0001
(Int1)(Int4)	1.396e+11	25	5.585e+09	1.269	0.1695
(Int2)(Int3)	1.072e+12	25	4.287e+10	9.742	0.0000
(Int2)(Int4)	1.436e+11	25	5.744e+09	1.305	0.1441
(Int3)(Int4)	2.355e+11	25	9.419e+09	2.140	0.0009

Summarizing all results of the *Additional file* and the *Text*, it is very difficult to determine precise conditions of convergence, especially if a system has a high degree of freedom.

References

1. Voit EO, Almeida JS: **Decoupling dynamical systems for pathway identification from metabolic profiles.** *Bioinformatics* 2004, **20:** 1670-1681.
2. Epureanu BI, Greenside HS: **Fractal basins of attraction associated with a damped Newton's method.** *SIAM Rev* 1998, **40:** 102-109.
3. Kikuchi S, Tominaga D, Arita M, Takahashi K, Tomita M: **Dynamic modeling of genetic networks using genetic algorithm and S-system.** *Bioinformatics* 2003, **19:** 643-650.
4. Schwacke JH, Voit EO: **Computation and analysis of time-dependent sensitivities in Generalized Mass Action systems.** *J Theor Biol* 2005, **236:** 21-38.
